# The effect of 0.1% topical ciclosporin a for 12-weeks on the ocular surface innate and adaptive immune response in dry eye disease: protocol for a randomized controlled clinical trial

**DOI:** 10.1080/07853890.2026.2729947

**Published:** 2026-09-19

**Authors:** Soumen Sadhu, Isabelle Jalbert, Mengliang Wu, Farshid Karimi, Ali Alghamdi, Laura E. Downie, Fiona Stapleton

**Affiliations:** a School of Optometry and Vision Science, UNSW Sydney, Sydney, NSW, Australia; b Department of Optometry and Vision Sciences, The University of Melbourne, Parkville, Victoria, Australia

**Keywords:** Dry eye disease, ciclosporin A, ocular surface immune cells, *in vivo* confocal microscopy, impression cytology

## Abstract

**Background:**

Ocular surface immune cells and pro-inflammatory cytokines have been reported to be increased in Dry eye disease (DED). Immunosuppressive agents such as topical cyclosporine A (CsA) can inhibit dendritic cell and T cell activation. However, CsA’s impact on ocular surface immune responses and tear cytokines has not been systematically studied. This paper describes a clinical trial protocol to investigate the effects of 0.1% CsA plus mineral oil eye drops, versus mineral oil eye drops alone on the ocular surface immune response in DED.

**Methods:**

This two-arm examiner-masked randomized controlled trial will enrol 60 eligible participants with moderate-to-severe DED. Participants will be randomized (1:1) either into the CsA plus mineral oil eye drops or mineral oil eye drops alone group and evaluated at baseline, 4-weeks and 12-weeks. Corneal immune cells will be assessed using static *in vivo* confocal microscopy (IVCM) for total corneal epithelial immune cells and with a novel technique, functional-IVCM (Fun-IVCM), to differentiate corneal epithelial immune cell subsets (i.e. putative T cells from dendritic cells). Conjunctival immune cells will be phenotyped using impression cytology and flow cytometry (CD45, CD3, CD4, CD8, CD11c, HLA-DR). Tear cytokine analyses will be conducted for IL-2, IL-4, IL-6, IL-12, IL-17, IL-23, IFN- γ using a multiplex bead assay.

**Conclusion:**

The clinical trial combines ocular intravital imaging, immune cell phenotyping and cytokine analysis to advance knowledge on the application of CsA in moderate-to-severe DED. It may also establish new immune cell parameters as biomarkers for monitoring the efficacy of anti-inflammatory DED treatments. *Trial registration:* ClinicalTrials.gov, NCT06898853.

## 1. Introduction

### 1.1. Background

The immune system plays an important role in the pathogenesis of dry eye disease (DED) [1]. Altered tear homeostasis leads to an increase in tear osmolarity and promotes the activation of inflammatory pathways in the ocular surface epithelia [1–3]. Changes to the ocular surface in DED include increased level tear cytokines, and chemokines, the expression of HLA-DR molecules and changes to immune cells in the conjunctiva [3,4]. Increased expression of CD4+ and CD8+ T cells in the ocular surface tissues of DED patients suggests a potential role of adaptive immunity in the chronicity of DED [3–9]. The presence of corneal and conjunctival antigen-presenting cells (APC) in the cornea and conjunctiva, and the upregulation of MHC II, IL-2, IL-17, IL-6 and IFN- γ and other pro-inflammatory molecules, in the tears of DED patients is also consistent with local adaptive immune response [10–15].

Although the conjunctiva has proven a useful tissue for studying ocular surface immunity, assessment of corneal immune cell changes has been limited by the lack of suitable, non-invasive sampling methods [4,16,17]. Researchers have employed various techniques including impression cytology, and conjunctival biopsy, to study conjunctival immunology [16–18]. Flow cytometry is a powerful technique that allows for the phenotypic analysis of immune cells through immunolabeling of extracellular expressions of cellular markers. An increased expressions of activation markers like HLA-DR as well as various immune cell populations, including T cells subsets (CD4+ and CD8+), B cells and natural killer (NK) cells, have been observed in DED using flow cytometry on conjunctival impression cytology samples [4,6–8,19]. However, these methods are invasive and have limited application to the cornea, due to the inappropriateness of performing impression cytology on this delicate tissue. The advent of *in vivo* confocal microscopy (IVCM) has, enabled the non-invasive visualization of ocular surface epithelial immune cell (EIC) and corneal sensory nerves [20–26].

Over the past 20 years, clinicians and researchers have used IVCM to examine corneal pathologic changes in a range of ocular surface conditions. Several IVCM studies have described changes to the density and morphology of corneal immune cells in DED [26–29]. While the *in vivo* nature of IVCM prevents direct immune cell phenotyping, immune cells observed in the healthy human corneal epithelium were historically believed to comprise of only dendritic cells (DCs), but recent evidence identified a proportion of these cells as T cells [30,31]. This knowledge advance was made, in part, with the advent of a recently-pioneered, novel imaging technique, termed functional-*in vivo* confocal microscopy (Fun-IVCM) which enables identification of corneal immune cell subsets through the integrated evaluation of their morphology and dynamics (i.e. cell morphodynamics) [30,31]. Fun-IVCM involves the consecutive capture of corneal IVCM images, of the same tissue region, every few minutes over a 30-minute period. The time-lapsed images are then registered to static tissue landmarks (e.g. corneal nerves), to reveal the morphodynamics of distinct immune cell subsets. Based on their morphodynamic behaviour, cells can be putatively differentiated as epithelial T cells (highly motile, with a small cell body) or DCs (relatively sessile, with a large cell body and obvious dendrites) (Fun-IVCM video file: https://doi.org/10.6084/m9.figshare.31562428). In the corneal stroma, macrophages can also be identified by their characteristic cytoplasmic movement between stationary keratocytes. Time-lapsed Fun-IVCM imaging allows for the quantification of a range of immune cell motility parameters, such as instantaneous and displacement speeds [30,31]. The *in vivo* identification of these corneal immune cell subsets using Fun-IVCM was validated using a mouse model of corneal infection, where phenotyped corneal epithelial T cells, were characterized using intravital 2-photon microscopy combined with *ex vivo* immunohistochemical analyses of donor human corneas [30]. With this advancement in imaging, various corneal immune cell subsets that were difficult to differentiate can now be quantified, and their behaviour can be monitored longitudinally. As these immune cell subsets are defined *in vivo* based on morphodynamic features, in the absence of capacity to perform molecular phenotyping, the term ‘putative’ is used to acknowledge this inherent limitation in immune cell subset delineation using Fun-IVCM. Beyond cell identification, Fun-IVCM enables quantitative assessment of immune cell dynamics within the living human cornea. This includes measurement of morphodynamic parameters such as instantaneous and displacement speed, arrest coefficient, trajectory patterns and changes in cell morphology over time [30,31]. These dynamic features provide functional information on immune cell behaviour, including tissue surveillance, motility states and cell-cell interactions that cannot be captured using static imaging approaches. Therefore, Fun-IVCM provides a unique opportunity to investigate not only the density of corneal immune cells, but also their functional behaviour *in vivo*, which cannot be captured using conventional static IVCM or *ex vivo* immune profiling techniques. The present study implements this innovative approach to investigate, for the first time, the effect of topical ciclosporin A (CsA) immunosuppressive therapy on ocular surface immune cell subsets and characteristics in DED. This novel imaging approach is combined with, conjunctival immune cell phenotyping and tear cytokine profiling. Despite the established anti-inflammatory efficacy of CsA in DED, it remains unclear whether treatment induces measurable changes in specific ocular surface immune cell populations and their functional characteristics *in vivo.*

First-line treatment for DED involves lubricant eye drops. However, due to the chronic nature of the condition, additional interventions, including anti-inflammatory therapies are often required to achieve adequate disease control [32–34]. Many of the current therapeutic approaches target DED inflammatory pathways, aiming to break the so-called vicious cycle of DED [3,35,36]. Both topical corticosteroids and immunosuppressants can reduce inflammation in DED [33,34,37]. Topical corticosteroids are used to manage acute exacerbations of inflammation, but their longer-term use is limited by their potential side-effects which include raised intra-ocular pressure, cataract formations and increased risk of ocular infections. In contrast, immunosuppressants agents such as ciclosporin A (CsA) can induce anti-inflammatory effects, with fewer adverse ocular side-effects [32–34]. Topical CsA treatment in chronic DED has been shown to significantly reduce ocular surface staining and ocular redness and improve tear volume and DED symptoms [38–41]. CsA dampens T cell proliferation by inhibiting the phosphatase calcineurin [42]. It functions at different steps of the immune response, during antigen presentation and DC-T cell interaction. Innate immune cells are now seen as potentially important targets of CsA and its mode of action may involve simultaneous targeting of DCs, macrophages and neutrophils [42]. At the ocular surface, there is preliminary evidence for CsA reducing activation markers such as HLA-DR, CD11c and certain apoptosis markers and conjunctival T lymphocytes, in patients with DED [43–47].

While several clinical trials and, systematic reviews have studied the efficacy of topical CsA for reducing ocular surface inflammation and improving signs and symptoms of DED [38,46–53], the effect of CsA on ocular surface immune cells has not been fully investigated using *in vivo* imaging techniques and immune cell phenotyping. Given the potential effects of CsA on both innate and adaptive immune cell subsets, we hypothesize that the use of topical 0.1% CsA will attenuate the ocular surface immune response and lead to an improvement in DED signs and symptoms. This study will address a significant evidence gap by investigating the effect of topical CsA on corneal and conjunctival immune cells using advanced clinical imaging. Further, it will investigate the effect of CsA on DCs (CD11c + DCs, HLA-DR) and T helper cell populations (CD4+ T cells) through analysis of lymphocyte markers (CD45, CD3, CD4, CD8) using flow cytometry on conjunctival impression cytology samples. The associated cytokines (Th1 pathway cytokines), particularly IL-2, IFN- γ and IL-17 will also be assessed in tear samples, which are primary targets of the immunomodulatory actions of CsA. This will be the first randomized controlled clinical trial to longitudinally assess both innate and adaptive immune cell characteristics using a multimodal approach at the ocular surface in DED participants undergoing CsA therapy. This integrated approach enables exploration of immune biomarkers of DED that could predict or explain therapeutic responses. The results of the clinical trial will address a clinically relevant question of whether adding topical 0.1% CsA along with a standard lubricant eye drops regimen produces measurable changes in ocular surface immune responses compared to lubrication alone.

### 1.2. Study aims and hypothesis

*Aim 1:* To investigate and compare the effect of 12-weeks of topical 0.1% CsA therapy plus mineral oil eye drops (lubricating eye drop, mineral oils + glycerin) relative to mineral oil eye drops alone, on ocular surface innate and adaptive immune cells and tear cytokines in DED.

*Hypothesis 1:* Topical 0.1% CsA will attenuate inflammation at the ocular surface, as indicated by a reduction in both innate and adaptive immune cell density and morphodynamics and reduced conjunctival expression levels (CD45, CD3, CD4, CD8, CD11c, HLA-DR) and lower levels of pro-inflammatory tear cytokines (IL-2, IL-4, IL-6, IL-12, IL-17, IL-23, IFN- γ) in DED.

*Aim 2:* To investigate and compare the effect of 12-weeks of topical 0.1% CsA therapy plus mineral oil eye drops (lubricating eye drop, mineral oils + glycerin) relative to mineral oil eye drops alone on clinical DED signs and symptoms.

*Hypothesis:* 0.1% CsA will improve ocular surface staining, tear film break-up time, tear production and DED symptoms.

*Aim 3:* To investigate the correlation between DED signs, symptoms and with corneal and conjunctival immune cell characteristics and tear cytokines levels in DED

*Hypothesis:* A higher corneal and conjunctival immune cell density (DC, T lymphocytes, HLA-DR positivity) and higher concentrations of pro-inflammatory tear cytokines (IL-2, IL-4, IL-6, IL-12, IL-17, IL-23, IFN- γ) correlate with worse clinical DED signs (reduced TBUT and tear volume, increased ocular surface staining) and symptoms.

*Aim 4:* To investigate the temporal relationship between conjunctival and corneal immune cell changes and alterations in clinical signs and symptoms of DED following treatment with topical 0.1% CsA.

*Hypothesis:* Ocular surface immune cell changes such as decrease in corneal immune cell density and decrease in conjunctival immune cell expressions following treatment with 0.1% CsA precede clinical improvements in signs and symptoms of DED.

## 2. Methods

This protocol is reported in accordance with the Standard Protocol Items: Recommendations for Interventional Trials guidelines (SPIRIT checklist, Supplemental File S1). The study was prospectively registered on ClinicalTrials.gov (NCT06898853) on 2 April 2025. Study interventions are listed on the Australian Therapeutic Goods Administration – Australian Registry for Therapeutic Goods (ARTG) for the treatment of DED – as Ikervis^®^ – Ciclosporin A 0.1% eye drops – unit dose-unpreserved (ARTG ID 319502), Cationorm multi dose preservative free lubricant eye drops 10 mL (ARTG ID 320453) and Refresh Plus – carboxymethylcellulose sodium 0.5% – unit dose – unpreserved lubricant eye drops (ARTG ID 127559). The clinical trial is sponsored and insured by the University of New South Wales, Sydney, Australia. Ethics approval was obtained from the University of New South Wales, Human Research Ethics Committee number iRECS7617 (UNSW IIT Protocol 202412001, version 4; date: 20 January 2025).

### 2.1. Trial design

An investigator-initiated, examiner-masked, two-arm randomized, parallel-group clinical trial was designed to evaluate the effect of topical 0.1% topical CsA plus mineral oil eye drops, compared to mineral oil eye drops alone on ocular surface immune cells, tear cytokines and clinical signs and symptoms of DED. The study design is depicted in Figure 1. The trial follows a superiority framework, aiming to determine whether adding topical 0.1% CsA is superior to mineral oil lubricating eye drops in improving the pre-specified outcomes. Eligible participants will be allocated randomly in a 1:1 ratio to one of the following intervention groups:

**Figure 1.**
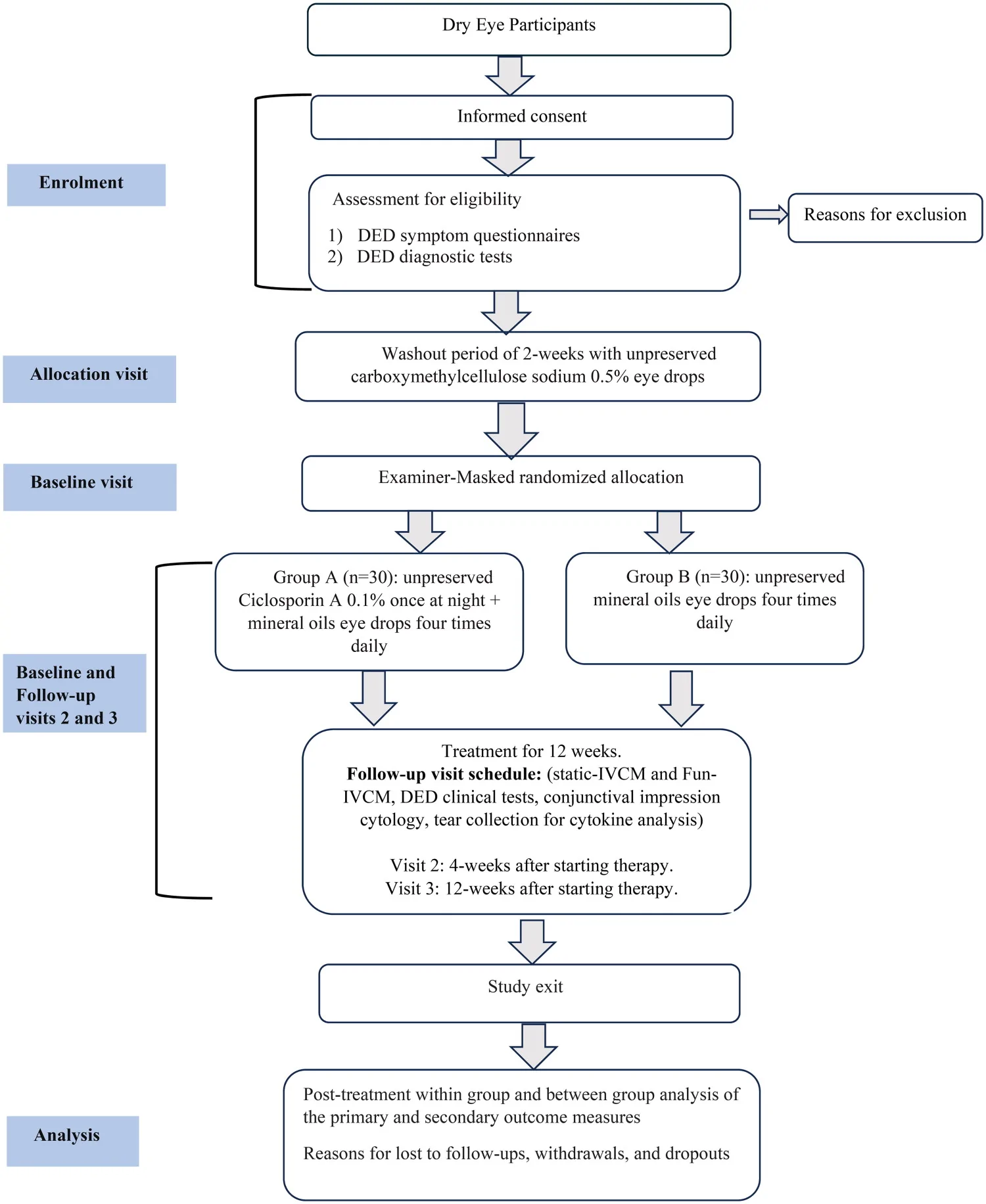
A schematic diagram of the trial design.

**Group A –** Topical unpreserved CsA 0.1%, one drop once daily at night-time + mineral oil eye drops (unpreserved lubricant eye drop: mineral oils + glycerin), one drop, four times daily in both eyes for 12-weeks.

**Group B –** Mineral oil eye drops alone (unpreserved), one drop four times daily in both eyes for 12-weeks.

### 2.2. Outcome measures

The co-primary outcome measures will be the change from baseline, at 4-weeks and 12-weeks within and between groups for: (i) total epithelial immune cell density in the central and corneal whorl region derived from static IVCM; (ii) putative epithelial T cell, DC densities in the corneal whorl region derived from Fun-IVCM. A full description is presented in Table 1.

**Table 1.** Co-primary outcome measures – change from baseline in Static-IVCM and, Fun-IVCM parameters and conjunctival immune cell populations at 4-weeks and 12-weeks post-treatment.

Outcome measures	Description	Time points
Static-IVCM (central and corneal whorl region)		
Total epithelial immune cell density (cells/mm^2^) (the sum of putative T cells, DCs in the corneal epithelium)	Number of cells per 1 mm^2^ tissue area. Higher immune cell density is thought to indicate higher immune response on the ocular surface [54].	Baseline, 4-weeks and 12-weeks.
Fun-IVCM (corneal whorl region)		
Putative epithelial T cell density (cells/mm^2^), putative epithelial DC density (cells/mm^2^)	Number of cells per 1 mm^2^ tissue area. Higher density values are thought to indicate greater immune response on the ocular surface [30,55].	Baseline, 4-weeks and 12-weeks.

Key secondary outcome measures will comprise the change from baseline, at 4-weeks and 12-weeks within and between groups for: (i) expression levels of conjunctival immune cell populations (% of positive cells for each of, CD45, CD3, CD4, CD8, CD11c and HLA-DR); (ii) corneal immune cell dynamics (putative T cell instantaneous speed, displacement speed and DC dSEARCH index, probing speed and others as detailed in Table 1); (iii) corneal immune cell morphology (for each of putative T cell, DCs and macrophages as detailed in Table 2); (iv) tear cytokine concentrations (IL-2, IL-4, IL-6, IL-12, IL-17, IL-23, IFN- γ, in pg/mL); (v) Ocular Surface Disease Index (OSDI) score; (vi) numerical rating scale of individual ocular surface symptoms; (vii) Oxford staining scores (corneal and conjunctival); (viii) non-Invasive tear film break-up time; (ix) fluorescein tear film break-up time; (x) tear meniscus height; (xi) meibomian gland dysfunction grade; (xii) conjunctival hyperaemia grade and xiii) Incidence of treatment-related adverse events. A detailed description is presented in Table 2.

**Table 2.** Key secondary outcome measures- change from baseline in corneal immune cell morphology, tear cytokines and DED signs and symptoms, at 4-weeks and at 12-weeks post-treatment.

Outcome measure	Description	Time points
Conjunctival impression cytology (superior and inferior bulbar conjunctiva)		
CD45, CD3, CD4, CD8, CD11c and HLA-DR	Expression levels (% of positive cells) Higher expression levels are thought to indicate higher immune response on the ocular surface [3].	Baseline, 4-weeks and 12-weeks.
Corneal whorl region stromal macrophage density (cells/mm^2^)	Number of cells per 1 mm^2^ tissue area. Higher immune cell density is thought to indicate higher immune response on the ocular surface [54].	Baseline, 4-weeks and 12-weeks.
Dynamic parameters for corneal epithelial putative T cells and putative stromal macrophages [31]		
Mean instantaneous speed (µm/min) (only epithelial putative T cells)	Averaged instantaneous speeds, calculated by the track length between two consecutive time-points, divided by time interval in µm/min. Higher values tend to indicate a heightened pro-inflammatory state [30,55].	Baseline, 4-weeks and 12-weeks.
Displacement speed (µm/min) (only epithelial putative T cells)	Net cell displacement length from first to last timepoint, divided by total time in µm/min.	Baseline, 4-weeks and 12-weeks.
Meandering index (only epithelial putative T cells)	Net cell displacement/total track length	Baseline, 4-weeks and 12-weeks.
Dancing index (only putative stromal macrophages)	Calculated as a percentage derived from a calculation of the nonoverlapped cell area divided by the total (summed) area, after colocalizing the same macrophage cell across two consecutive time intervals (based on aligning the centroids of the two cell shapes), normalized to time, and then averaged across all time intervals for a given macrophage	Baseline, 4-weeks and 12-weeks.
Dynamic parameters for corneal epithelial DCs [31]		
dSEARCH index	Cumulative distance of dendrite extension and retraction of all dendrite tips, from a single cell, divided by time	Baseline, 4-weeks and 12-weeks.
DC probing speed (µm/min)	Total displaced length of each dendrite tip/total time, averaged for all dendrite tips from one cell measured in µm/min Higher values tend to indicate a heightened pro-inflammatory state [30,55].	Baseline, 4-weeks and 12-weeks.
Tear cytokine concentrations		
IL-2, IL-4, IL-6, IL-12, IL-17, IL-23, IFN- γ	Concentrations in the tear fluid. Measured in pg/mL Higher values tend to indicate a heightened pro-inflammatory state [14,56]	Baseline and 12-weeks.
Morphological parameters for corneal epithelial putative DCs, T cells and stromal macrophages [31]		
Cell area (µm^2^)	Area within a freehand tracing around the cell boarder. Higher values tend to indicate a heightened pro-inflammatory state [30,55].	Baseline, 4-weeks and 12-weeks.
Cell perimeter (µm)	Total length of the freehand tracing around the cell border measured in µm Higher values tend to indicate a heightened pro-inflammatory state [30,55].	Baseline, 4-weeks and 12-weeks.
Cell circularity	4π x (cell area)/(perimeter)^2^	Baseline, 4-weeks and 12-weeks.
Cell solidity	Area of the cell divided by the convex hull area.	Baseline, 4-weeks and 12-weeks.
Cell field area (µm^2^) (only for DCs)	Area of the polygon formed by connecting the dendrite tips of the cell	Baseline, 4-weeks and 12-weeks.
Dendritic complexity (number) (only for DCs)	The number of dendrite tips per cell	Baseline, 4-weeks and 12-weeks.
Clinical DED parameters (signs and symptoms)		
Oxford staining scores (cornea and conjunctiva)	Extent of corneal fluorescein and conjunctival lissamine green dye staining using a 6-point Oxford grading scale from 0–5, in an increment of 1 unit per step. The nasal and temporal conjunctiva and the cornea will be each be scored from 0–5, giving a total score range from 0–15. Higher scores indicate greater epithelial damage (clinical cutoff ≥1) [57,58]. Arbitrary units.	Baseline, 4-weeks and 12-weeks.
Ocular Surface Disease Index (OSDI) score	Severity of DED symptoms, scored from 0 to 100, in an increment of 1 unit, where higher scores indicate greater symptom severity (clinical cutoff ≥13) [59]. Arbitrary units.	Baseline, 4-weeks and 12-weeks.
Non-invasive tear film break-up time	Tear film stability defined in seconds, measured using the Keratograph 5 M device, in an increment of 0.01 s. Three measurements per eye will be averaged. A value of >10 s is considered normal and lower values indicates more severe disease [60]. (clinical cutoff <10 s).	Baseline, 4-weeks and 12-weeks.
Fluorescein tear film break-up time	Tear film stability defined in seconds, measured after fluorescein dye instillation and observing the appearance of first break (black spot) of the tear film on the cornea using a cobalt-blue filter in a slit-lamp. Measured in seconds in an increment of 1 s. Three measurements per eye will be averaged. A value of >10 s is considered normal and lower values indicates more severe disease [60]. (clinical cutoff < 10 s).	Baseline, 4-weeks and 12-weeks.
Numerical rating scale for symptoms	Severity of specific DED symptoms for dryness, vision quality, fluctuating vision, itching, burning sensation, foreign body sensation, eye pain and overall discomfort will be assessed using a scale, ranging from 0–100, in an increment of 1 unit, where lower scores indicate higher symptom severity [61]. Arbitrary units.	Baseline, 4-weeks and 12-weeks.
Conjunctival hyperaemia	Defined as the intensity of redness graded on a 5-point scale from 0–4, in an increment of 0.01 unit, measured with the Keratograph 5 M device, averaged for nasal and temporal conjunctiva, where higher scores indicate higher hyperaemia. Arbitrary units.	Baseline, 4-weeks and 12-weeks.
Tear meniscus height	Measure in mm, using the Keratograph 5 M device under infrared light at the 6 o’ clock position, in an increment of 0.01 mm, averaging three readings per eye. Values >0.20 mm considered normal and lower values indicates more severe disease (clinical cutoff <0.20 mm) [60].	Baseline, 4-weeks and 12-weeks.
Meibomian gland dysfunction	Meibomian Gland Yielding Liquid Secretion (MGYLS) assessed over 15 lower eyelid glands, with each scored as 0 (no secretion) or 1 (secretion), for a total score of 0–15 per eye (clinical cutoff ≥5) [62]. Lower scores indicate more severe disease. Meibomian Gland Score (MGS) to grade secretion quality across the same 15 glands: with 0 = clear, 1 = cloudy, 2 = cloudy with particulates, 3 = toothpaste-like; total score 0–45 per eye (clinical cutoff ≤12) [63]. Lower scores indicate more severe disease.	Baseline, 4-weeks and 12-weeks.
Incidence of treatment-related adverse events	Proportion of participants experiencing adverse events judged by the investigator as related to study treatment.	4-weeks and 12-weeks.

Exploratory outcome measures will comprise the change from baseline, at 4-weeks and 12-weeks within and between groups for: (i) central and temporal peripheral corneal nerve length; (ii) central and temporal peripheral corneal nerve density; (iii) central and temporal peripheral corneal nerve tortuosity; (iv) other corneal nerve morphological features such as beading and micro neuromas and iv) neuro-immune interactions (i.e. interactions between corneal nerves and each of putative T cells and DCs).

### 2.3. Participant eligibility criteria

Inclusion Criteria

1. 18 years of age and above with persistent DED symptoms for 6 months or more.
2. Currently using artificial tear eye drops for DED
3. OSDI score of ≥23 (moderate and higher DED symptoms), and all these following DED clinical signs in at least one eye: i) Oxford staining score of ≥1 either in the cornea or conjunctiva ii) Tear meniscus height < 0.2 mm iii) non-invasive tear film break-up time of <10 seconds.

Exclusion Criteria

1. Current use or previous use in the last 6 months of corticosteroids, ciclosporin, lifitegrast or any anti-inflammatory eye drops.
2. Current use of systemic CsA or tacrolimus.
3. Known hypersensitivity or contraindication to the study interventions or any of their ingredients or agents used in the study.
4. Active intraocular inflammation.
5. Current contact lens wear or the use of contact lenses in the last 4-weeks.
6. Active eye infections.
7. A history of critical illness.
8. DED secondary to Steven-Johnson syndrome and cicatricial conjunctival disease.
9. Presence of other ocular co-morbidities.
10. Current use of any glaucoma medications.
11. A history of ocular surgery in the past 6 months.
12. Any other active or inactive systemic condition, structural abnormality such as an eyelid malposition, which is in the judgment of the investigator could confound the study assessments or limit adherence, or compromise safety.
13. Women who are pregnant or breastfeeding.

### 2.4. Study intervention

Participants eligibility will be assessed by the investigators (S.S, A.A) after providing written informed consent (Supplemental File S2). Participants using any current lubricating eye drops, topical and oral medications for DED, and other DED-related interventions will be asked to discontinue their use and will then enter a 2-week wash-out period. Participants will be assigned carboxymethylcellulose sodium 0.5% (Refresh Plus, Allergan Aesthetics, an AbbVie company, Sydney, Australia) lubricating eye drops, for use in both eyes up to six times daily over the study period. After the 2-week wash-out period, participants will return to the clinic (baseline visit) and will be randomized (1:1) either into Group A or Group B. **Group A** will receive topical CsA 0.1% (Ikervis^®^ 1 mg/mL, manufactured by: Santen Pharmaceutical Asia Pte Ltd and distributed by: Seqirus (Australia) Pty Ltd) unpreserved unit dose ampules for use once daily at night along with oil eye drops (lubricant: combination of mineral oils + glycerin eye drops (Cationorm**^®^**, manufactured by- Santen Pharmaceutical Asia Pte Ltd & distributed by: Seqirus (Australia) Pty Ltd) 10 mL unpreserved bottle four times a day; **Group B** will receive mineral oil eye drops alone (Cationorm^®^ eye drops) four times daily. Participants will be asked to discontinue any DED medications, and management during the study period. Participants must meet the eligibility criteria both at eligibility screening and baseline visits. After initiation of the intervention, participants will return to the clinic for follow-up visits at 4-weeks and 12-weeks. A summary of the study design, constructed as per the CONSORT 2010 guideline is provided in Figure 1.

### 2.5. Randomization and allocation

Eligible participants (*n* = 60) will be randomly allocated to one of the two study intervention groups (A or B) in a 1:1 allocation ratio based on a pre-determined computer-generated randomization sequence by external personnel (not a member of the investigator group). Block randomization (block size of 6) will be used to ensure allocation balance. Investigators – S.S & A.A will be enrolling participants and be masked to the study treatment group. The interventions will be packaged in sealed identical opaque boxes and will be sequentially labelled (A or B) by external personnel as per the randomization list. Each box will have instructions of the medicine use, a leaflet detailing about potential side-effects and a sufficient amount of dosage for the treatment period. Participants will be instructed to use unpreserved ampules of 0.1% CsA only once in both eyes and not to re-use the same ampule again. For unpreserved mineral oil eye drops participants will be instructed to use the bottle for a month as per manufacturer guideline. The external personnel will be responsible for maintaining the randomization list and will be the only person having access to the randomization list. As this is an examiner-masked clinical trial, study investigators will remain masked to the treatment groups. The external personnel will be responsible for handling and distributing the investigational product boxes to participants. An investigator involved in clinical assessments will explain the masking process to participants and instruct them not to disclose which medication they are using to the study investigators. Each medication box will also include a leaflet explaining the importance of masking, along with instructions on how to contact the external personnel if participants require assistance regarding the study medication. Whenever necessary, the principal investigators (F.S and I.J) can perform an emergency decoding and breaking of the blind for individual participants. Following randomization, study follow-up visits will be conducted at 4-weeks and 12-weeks. Study procedures at each visit will be performed in a set sequence. Participants will be instructed not to use their assigned investigational product for at least 2–4 h prior to each visit. For each follow-up, safety measures such as best-corrected visual acuity, intra-ocular pressure and post procedures corneal staining will be assessed, and a dairy and used medicine ampules/bottles will be collected for adherence monitoring. The order of study procedures is provided in Table 3.

**Table 3.** Sequence of study procedures at each follow-up visit.

	Study period			
	Enrolment	Randomized allocation	Post-allocation	
Procedure	− 2 weeks (Eligibility screening)	Baseline	4-weeks	12-weeks
Informed consent	X			
History (ocular and systemic)	X			
Ocular Surface Disease Index Questionnaire (OSDI) and numerical rating scale for symptoms questionnaire	X	X	X	X
Adverse events query		X	X	X
Eye drops usage query		X	X	X
Collection of adherence diary and used/unused ampules/bottles		X	X	X
Best-corrected visual acuity (bilateral)	X	X	X	X
Keratograph 5 M (bilateral): NIBUT, TMH, Conjunctival hyperaemia	X	X	X	X
Tear collection with Schirmer strips (bilateral)		X	X	X
Slit-lamp examination (bilateral): ocular surface assessment, Fluorescein tear film break-up time, ocular surface staining evaluation, meibomian gland evaluation	X	X	X	X
Wash-out period with unpreserved ocular lubricant	X			
Corneal *in vivo* confocal microscopy (Static and Fun-IVCM) (unilateral – right eye only)		X	X	X
Conjunctival impression cytology collection (unilateral – right eye only)		X	X	X
Intra-ocular pressure (bilateral)		X	X	X
Dispense study investigational products		X	X	
Collection of unused study investigational products		X	X	X

NIBUT: non-invasive tear break-up time; TMH: tear meniscus heigh; IVCM: *in vivo* confocal microscopy.

### 2.6. Recruitment

Participants will be recruited from the University of New South Wales optometry clinic *via* printed advertisements, posters and flyers and from the community (*via* a recruitment letter, (Supplemental File S3). Email invitations will be sent to participants from the optometry clinic database who have previously expressed interest in research participation. Further online advertisements through Facebook, newspapers and other local social media groups will be initiates as required. Study advertisements will be distributed through collaborating with local hospitals and private clinics, particularly optometrists, ophthalmologists, immunologists and rheumatologists. Advertisements may also be distributed by relevant organizations such as Arthritis Australia and the Australian and New Zealand Society of Immunology.

### 2.7. Corneal IVCM for immune cell quantification

IVCM (Heidelberg Retinal Tomograph III with Rostock Corneal Module, Germany) will be used to assess corneal immune cell and nerve parameters. **Static-IVCM** will be performed in the right eye to quantify epithelial immune cell density and corneal nerves at 4 corneal tissue locations: central cornea, inferior whorl, inferior peripheral cornea and temporal cornea after application of unpreserved topical anaesthetic (oxybuprocaine hydrochloride 0.4%, Minims, Bausch.co.uk). A sterile disposable Perspex cap (Tomo-cap; Heidelberg, Germany) will be attached in front of the objective lens and a carbomer gel (Viscotears, Alcon, NSW, Australia) will be used as a coupling agent. Imaging of the corneal sub-basal nerve plexus will occur at a tissue depth of 25–65 µm from the superficial epithelium, where the immune cells are visible; approximately 50–100 section images will be captured at each tissue location.

**For Fun-IVCM** sequence and volume (z-stack) scans [400(x) × 400(y) × 80(z) μm] will be captured spanning the basal epithelium and anterior stroma, at the corneal whorl and ∼2 mm inferior (peripheral) to the whorl, over about a 25 to 30 min, with intervals of about 5 ± 1.5 min, between consecutive image captures, per the scanning protocol in Supplemental Figure S4. A single investigator (S.S or A.A) masked to the study timepoints, and study group assignment will manually analyze the images. The total number of epithelial immune cells in a 400 × 400 µm frame representing a tissue area of 160,000 µm^2^ will be manually counted, and their density will be presented in cells/mm^2^. For Fun-IVCM, 80–100 section images from each sequence scan will be stitched into a montage image, followed by the creation of a time-lapsed video through registration of the montage images obtained at different scanning timepoints, using a customized MATLAB script [30,31,64]. Individual time-lapsed videos will be created for the basal epithelium at the corneal whorl, the basal epithelium in the peripheral corneal region, and the anterior stroma at the whorl. The time-lapsed Fun-IVCM videos (Fun-IVCM video file: https://doi.org/10.6084/m9.figshare.31562428) allow for identification of corneal putative DCs (relative stationary with dendrite tip probing), T cells (motile with a small cell body) and anterior stromal macrophages (relative stationary with an ameboid movement), and for quantification of cell motility parameters (described in Table 2).

### 2.8. Conjunctival impression cytology and tear collection using Schirmer strips

Superficial conjunctival epithelial cells will be collected by applying single use polyethersulfone filters (0.2 µm pore, 13 mm; Supor Membranes, Gelman Sciences, MI) to the superior and inferior bulbar conjunctiva for 2–4 s after topical anaesthetic [8,65]. Visible cell patches should cover >50% of the filter surface. Filters will be transferred to tubes with 2 mL PBS containing 0.05% paraformaldehyde, and cells will be dislodged by gentle agitation/scraping with a micropipette tip. Suspensions will be centrifuged (1600 rpm, 5 min), supernatant discarded, and cells will be stained with 2–3 µL (titrated) each of CD45, CD3, CD4, CD8, CD11c and HLA-DR antibodies (BD Biosciences) in PBS with 1% BSA. After 30 min incubation at 4 °C in the dark, samples will be washed twice with PBS, resuspended in 100 µL PBS, and acquired on a Cytek Aurora flow cytometer using appropriate gating strategy. Compensation will be set with fluorescence beads, with negative and positive controls prepared before acquisition. Periodic controls, unstained samples and fluorescence minus one (FMO) control will be included throughout the experiment to monitor staining consistency and assist with accurate gating. During initial panel setup and optimization, peripheral blood mononuclear cells (PBMCs) will be used as a positive control to validate antibody performance, confirm population separation, antibody titration and establish gating boundaries for each marker. The gating strategy involves initial exclusion of debris and non-cellular events using a forward scatter area (FSC-A) versus side scatter area (SSC-A) gate, followed by FSC-H versus FSC-A singlet gating to remove doublets and cellular aggregates (Supplementary Figure S5). Following singlet gating, CD45 (BUV395) is used to identify CD45+ leukocytes, which serve as the parent population for downstream immune phenotyping [5,9,17,65–67]. CD45+ cells are gated on CD3 (BV480) to identify CD3+ T cells and CD3− populations. The CD3− population is gated on CD11c (PE) to identify CD11c + dendritic cell populations (CD45 + CD3 − CD11c+). CD3+ T cells are further gated on CD4 (FITC) versus CD8 (BUV805) to identify CD4+ helper T cells and CD8+ cytotoxic T cell subsets. Expression of the activation marker HLA-DR (R718) is then assessed within each identified immune cell population, including CD45 + HLA-DR+, CD3 + HLA-DR+, CD4 + HLA-DR+, CD8 + HLA-DR+ and CD11c + HLA-DR+ cells.

For tear collection, Schirmer strips will be placed at the lateral canthus for 5 min, wetting length will be recorded and then strips will be stored in Eppendorf tubes at −80 °C until tear extraction for the cytokine analysis, in accordance with the pre-analytical handling recommendations [68,69]. Tear proteins will be extracted by elution through centrifugation and cytokine concentrations (IL-2, IL-4, IL-6, IL-12, IL-17, IL-23, IFN-γ) will be quantified using a multiplex bead immunoassay and will be as raw concentrations in pg/mL [68,69]. Normalization, if possible, to total tear protein content or migration length will be decided based on the wetting lengths characteristics of the DED cohort at the end of the study and as per established protocols [68,69]. Schirmer strip wetting length will be recorded at each visit and may be included as a covariate in statistical analyses where normalization could not be suitable.

### 2.9. Sample size

The study sample size was determined based on the co-primary outcome, total epithelial central corneal immune cell density (i.e. the sum of putative T cells, DCs in the corneal epithelium). Using G*Power 3.1 and a repeated measures ANOVA with within-between interaction, the study is powered to detect minimum clinically significant change of 29 cells/mm^2^, which exceeds the day-to-day variation of 28 cells/mm^2^ in healthy eyes [70]. Assuming a standard deviation of 45 cells/mm^2^ (Cohen’s *f* = 0.25, medium effect size), with a significance level of 0.05, 80% power, and a 1:1 allocation ratio, a total of 54 participants is required. To account for a 10% dropout rate, the final sample size is set at 60 participants, with 30 per treatment group.

### 2.10. Data analysis

Analyses will be conducted in SPSS and R Studio. Continuous data will be tested for normality (Shapiro – Wilk, Q – Q plots) and will be reported as mean (SD) or median (IQR). The pre-specified co-primary outcomes will be analyzed using a hierarchical testing strategy to control the overall type I error rate. The analysis will first evaluate changes in total epithelial immune cell density in the central corneal region derived from static IVCM. Subsequent analyses of total epithelial immune cell density in the corneal whorl region derived from static IVCM, followed by putative epithelial T-cell and dendritic cell (DC) densities in the corneal whorl region derived from Fun-IVCM, will be considered confirmatory only if statistical significance is achieved for the preceding outcome(s) in the hierarchy. Corneal immune cell densities will be analyzed with a linear mixed-effects model (fixed: timepoint, treatment; random: participant), assessing main effects/interactions, adjusting for baseline and after checking residuals. The linear mixed-effects model will use all available repeated measurements under the missing-at-random assumption. Where the extent or pattern of missing data warrants further evaluation, multiple imputation will be undertaken as a sensitivity analysis. Further sensitivity analysis will be performed, based on the final data and results. Categorical outcomes will use GLMMs (binomial) with Type II Wald χ^2^; post hoc contrasts will be Tukey-adjusted on model-estimated marginal means. The same framework applies to conjunctival markers, tear cytokines and DED clinical signs/symptoms. For Aim 3, cross-lagged panel models (4–12 weeks) and mediation will test whether corneal immune-cell changes predict/mediate clinical improvement. Associations will be summarized with Pearson/Spearman coefficients and a correlation matrix; subgroups will be defined by baseline severity. Safety will be descriptive with AEs tabulated by system organ class. Statistical significance will be set at α = 0.05.

The study is powered based on the central corneal total EIC density, using a minimum clinically significant difference of 29 cells/mm^2^, which exceeds the reported day-to-day variability of 28 cells/mm^2^ in normals and 23 cells/mm^2^ in DED [70,71]. A day-to-day variation of 34 cells/mm^2^ has been reported for the corneal whorl region in DED [71] and therefore, any true biological treatment effect at either location would be expected to exceed these variability thresholds to be considered clinically meaningful. The study may be insufficiently powered to detect effects in secondary outcomes, including conjunctival immune cell phenotypes and tear cytokine concentrations.

### 2.11. Safety monitoring

A panel of safety indicators will be assessed at each visit, including best-corrected visual acuity using LogMar chart, intraocular pressure using iCare tonometer, slit-lamp examination of the anterior segment, and ocular surface staining after each of IVCM and impression cytology sampling. Safety assessments will evaluate the seriousness, causality and expectedness of any adverse events (AEs) in relation to the trial interventions. Known side effects of topical 0.1% cyclosporine A (CsA) for DED include transient instillation site pain, conjunctival hyperaemia, tearing, irritation, burning and stinging, reported in 12–37% of participants [39,40,46,47,72]. These are generally self-limiting and not associated with long-term pathology. The mineral oil lubricant eye drops may also cause transient burning or irritation in 11–21% of participants [39,40,46,47,72]. All adverse events, adverse reactions and treatment-emergent adverse events (TEAEs) will be recorded. All AEs and serious adverse reactions will be documented in a safety monitoring register. Any unexpected serious adverse reactions will be reported to the Human Research Ethics Committee and the Therapeutic Goods Administration (TGA, Australia) within 15 days. Compensation for harm resulting from trial participation will be provided in accordance with the Medicines Australia Guidelines for Compensation for Injury Resulting from Participation in Sponsored Clinical Trial, even though this is an investigator-initiated trial.

### 2.12. Monitoring adherence to treatment and protocol deviations

Treatment adherence of participants will be recorded using an adherence diary and through return of used/unused ampules/bottles at each visit. Protocol deviations will be documented in a separate log and will be reported to the principal investigator by the study investigator. Principal investigators will review the protocol deviation and the clinical trial protocol to establish the corrective actions and preventative steps to prevent the deviation from reoccurring. Participants may be permanently discontinued from the trial if it is deemed to be in their best interest by the study team. Criteria for discontinuation include persistent adverse events, adverse reactions and treatment-emergent adverse events that are not correctable, voluntary withdrawal by the participant, loss to follow-up, lack of adherence to interventions/visits or repeated protocol violations/deviations. For participants who discontinue the study for any reason, their last available follow-up data will be retained and included in the analysis.

### 2.13. Data handling

Study investigators will maintain accurate source documents and trial records for all observations. Each participant will be assigned a unique ID, and data will be recorded using case record forms. Access to study data will be restricted to the study investigators. The study investigators will have access to the final trial dataset. There are no contractual agreements in place that restrict the investigators’ access to the data. All data will be entered into the secure REDCap system, hosted by Research Technology Services at UNSW, Sydney. Data will be stored securely for 15 years following publication. All information will be handled in accordance with the Privacy and Personal Information Protection Act 1998 (NSW, Australia).

## 3. Discussion

This an examiner-masked randomized clinical trial capitalizes on the availability of novel imaging and laboratory techniques to comprehensively evaluate the immunomodulatory effects of 0.1% CsA plus mineral oil eye drops versus mineral oil eye drops alone in a well-characterized DED population. Key innovative approach combines corneal static IVCM and Fun-IVCM with conjunctival immune cell phenotyping and tear cytokine analysis, to provide comprehensive insights into how CsA affects both innate and adaptive immune responses in DED. The impact of CsA on the ocular surface immune response is poorly understood. While previous studies have demonstrated CsA’s beneficial effects on ocular surface signs and symptoms, its direct effect on specific immune cell populations remains incompletely characterized. The study aims to recruit participants with various aetiologies of DED including DED secondary to autoimmune disorders such as Sjögren disease. This study can elucidate CsA’s immunomodulatory mechanisms across a spectrum of inflammatory conditions in DED. This study is among the first to apply the novel Fun-IVCM technique to characterize putative T cells and dendritic cells in a clinical trial with the overlay of a therapeutic intervention. Further, apart from quantification of immune cell density, Fun-IVCM allows for the assessment of immune cell dynamic and morphological parameters, providing deeper insight into underlying ocular surface immune activity. Previous studies suggest that increased T cell density, along with higher instantaneous and displacement speed, may reflect higher immune surveillance and inflammatory activity [30,31]. The quantification of morphological parameters, such as DC cell area and perimeter, may also provide additional information regarding immune cell status and activation over time with treatment [26,30,54].

The use of anti-inflammatory therapies in DED is increasing as understanding of disease pathophysiology improves. However, there remains no single gold-standard diagnostic or monitoring biomarker for DED, and considerable efforts are ongoing to identify objective, reproducible markers with low inter- and intra-individual variability. Previous studies have showed that corneal intraepithelial immune cells exhibit relatively stable diurnal variation and studies have established day-to-day repeatability coefficients [70,71], supporting their potential utility as biomarkers. The current study will assess whether changes in immune cell density and morphodynamic parameters in response to treatment exceed established coefficients of repeatability values, thereby distinguishing true biological effects from physiological variability. A reduction in corneal and conjunctival T cell-associated parameters in the CsA group would be consistent with its known anti-inflammatory mechanism of action and may further support the role of ocular surface immune cell features as potential biomarkers for monitoring treatment response in DED (Figure 2).

**Figure 2.**
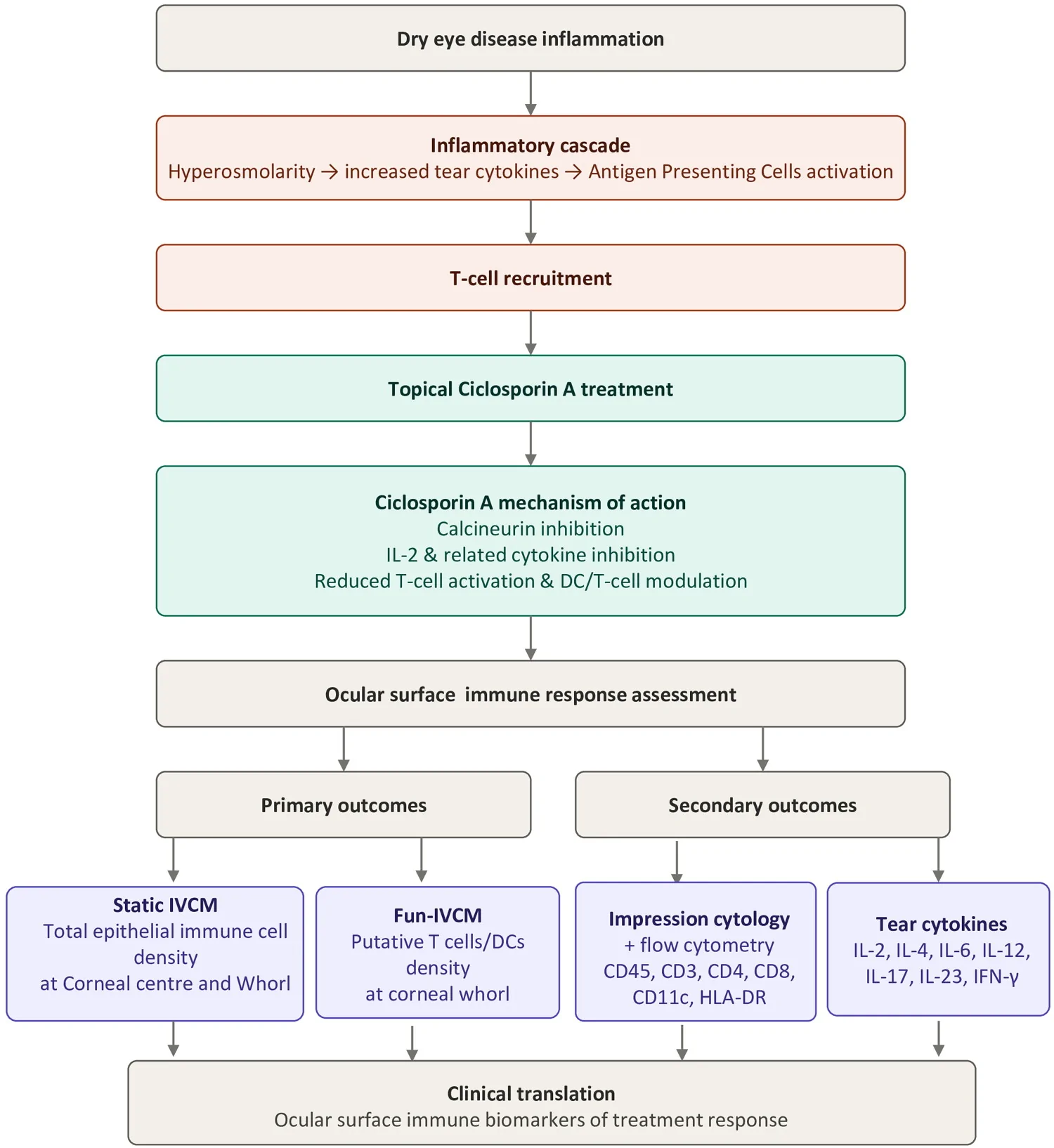
Schematic representation of dry eye disease associated ocular surface inflammation, the mechanism of action of topical ciclosporin A, and the outcome measures used to assess treatment related immune modulation of the ocular surface.

The study design has several key methodological considerations. First, the participant eligibility criteria balance the need to maintain clinical relevance and sufficient baseline inflammation to observe CsA effects, focusing on moderate to severe DED cases. The combination of inclusion criteria of tear meniscus height <0.20 mm, at least mild ocular surface staining – Oxford score ≥1, and moderate-to-severe symptoms – OSDI ≥23, is characterized by aqueous tear deficiency with at least mild-moderate level of inflammation. Overall, the study is expected to recruit a mixed type of DED. Second, the use of 0.5% carboxymethylcellulose sodium eye drops during the washout phase, allows for a cleaner evaluation of the active comparator effect during the study period. The mineral oils lubricant eye drops used in this trial are a cationic oil-in-water nanoemulsion formulation designed to enhance tear film stability and retention on the ocular surface, forming the vehicle base of topical ciclosporin A for Ikervis. As both treatment groups transition to the mineral oil eye drops simultaneously at randomization, any biophysical effects associated with this change will be equally distributed across study arms and therefore not confound the between-group comparison. Third, the mineral oil eye drops -controlled design, where both groups receive lubricant eye drops, reflects real-world clinical practice where CsA treatment is typically prescribed with lubricants as an adjunctive therapy. The multi-modal assessment of immune responses through Fun-IVCM, impression cytology and tear cytokine analysis will provide comprehensive data on CsA’s impact on the Th1 pathway and other immune mechanisms. Previous clinical studies have suggested that CsA’s therapeutic effects begin manifesting at approximately 4-weeks post-treatment initiation [38]. This study’s longitudinal design, with assessments at 4 and 12-weeks, will help characterize both the early and sustained immunomodulatory effects of CsA treatment. The detailed immune cell characterization may help identify specific biomarkers predictive of treatment response, in DED management.

The findings from this study have important clinical implications. Current DED management relies largely on symptom assessment and clinical signs, which may not always reflect underlying immune activity. Identification of objective immune biomarkers associated with therapeutic response could improve monitoring of treatment efficacy in DED management. Understanding the cellular immune changes associated with CsA treatment may help identify patients who are more likely to benefit from immunomodulatory therapy. Furthermore, the study aims to improve the understanding of the natural history of DED and ocular surface immune parameters with lubricant therapy compared with adjunctive CsA treatment. The study will form the baseline study for developing future study rationale and methods.

A limitation of the study is that, as an examiner-masked rather double-blind trial, participants will be aware of their group allocation, introducing a potential bias. However, the primary outcome measures – comprising corneal immune cell densities derived from static IVCM and Fun-IVCM, are objective, biological endpoints that we would not expect to be subjected to participant unmasking. Any between-group differences observed in these objective immune parameters at 4 and 12 weeks would therefore be attributable to the pharmacological action of the drug. The subjective outcomes – OSDI and NRS symptom scores, may be susceptible to bias, due to the absence of participant masking, and are designated as secondary outcome measures for this trial. The primary outcome measures are based on *in vivo* observation of immune cells, although Fun-IVCM provides unique insights into immune cell behaviour, immune cell classification remains putative because molecular phenotyping cannot be performed during *in vivo* imaging. Moreover, although the 12-week follow-up period is appropriate to assess early immunomodulatory effects of CsA, participants who respond slowly to the treatment may not be fully captured, and longer-term studies will be required to determine the effect on immune changes and their relationship with clinical outcomes. Future studies should validate these immune biomarkers in larger multicentre cohorts. Longer-term longitudinal studies will also be important to establish whether changes in immune cell characteristics precede clinical improvement and whether they can be used as surrogate markers for disease monitoring.

## Supplementary Material

Supplemental file S1 SPIRIT Checklist.doc

Supplemental figure S4 Fun IVCM protocol.png

Supplemental file S2 PARTICIPANT CONSENT FORM.pdf

Supplemental Figure S5 Gating strategy.png

Supplemental file S3 Recruitment Letter.pdf

## Data Availability

On completion of the trial, the full study protocol, de-identified participant-level dataset, will be made available upon reasonable request to the corresponding author, subject to ethics approval and data-sharing agreements.
