# Supplementary material for: The effect of 0.1% topical ciclosporin a for 12-weeks on the ocular surface innate and adaptive immune response in dry eye disease: protocol for a randomized controlled clinical trial
Source: Ann Med. 2026 Sep 19;58(1):2729947. doi: 10.1080/07853890.2026.2729947 (PMC13625527; doi:10.1080/07853890.2026.2729947)
Supplement: Supplementary file 1 [file IANN_A_2729947_SM8078.pdf]

School of Optometry and Vision Science

UNSW SYDNEY

Dear potential participant,

Researchers at UNSW are conducting a project to understand the effect of 0.1% cyclosporine A eye drops on immune cells at the surface of the eye in people with dry eyes.

Title: The effect of 0.1% ciclosporin A eye drops for 12-weeks on the eye surface immune cells in dry eyes

You are a potential participant for the study if you are

- Above 18 years of age
- You have signs and symptoms of or have already been diagnosed with dry eyes

You are not eligible to participate in the study if you meet any of the following conditions.

- You are currently using or have used any anti-inflammatory eye drops in the past 6 months.
- You are taking oral ciclosporin or tacrolimus.
- You have known allergy to the study medication or any of its ingredients.
- You have an active eye disease inside the eye.
- You are a regular contact lens user or have used contact lenses in the last 4 weeks.
- You have an active eye infection or critical illness
- You have dry eyes due to Steven-Johnson's syndrome, cicatricial conjunctival disease.
- You have other eye conditions or use eyedrops for glaucoma.
- You have a history of eye surgery in the past 6 months.
- You are pregnant or breastfeeding.

Participants will be asked to complete the following research activities if they agree to participate:

- Telephone and paper-based questionnaires will take approximately 2 minutes to complete.
- **The study involves 4 visits:**

  Visit 1: screening for eligibility (dry eye tests) and start 2-weeks wash-out period.

  Visit 2: at 2-weeks – dry eye tests, confocal microscopy, tear and cell collection. You will enter either into one of the two treatment arms
  <u>Group A</u> – Ciclosporin A (CsA) 0.1% once daily at night-time + Cationorm four times daily in both eyes for 12-weeks**.**
  <u>Group B</u> – Cationorm four times daily in both eyes for 12-weeks.

Visit 3: at 6-weeks – dry eye tests and confocal microscopy.

Visit 4: at 14-weeks - dry eye tests, confocal microscopy, tear and cell collection.

A full description of all research activities, including any risks, or discomforts that you may experience while participating in this research is included in the attached Participant Information Statement and Consent Form.

Please contact the following person via email or phone to register your interest in taking part in the research:

**Name**	Soumen Sadhu
**Position**	PhD candidate
**Telephone**	+614 21892071
**Email**	s.sadhu@unsw.edu.au

If you have questions about the research and would like to Investigator, Chief Investigator please contact the following person:

Chief Investigator

**Name**	Fiona Stapleton
**Position**	Scientia Professor
**Telephone**	+61 418868234
**Email**	f.stapleton@unsw.edu.au

Taking part in this research study is voluntary. You may choose not to take part. If you decide not to take part in this research, your decision will not affect your relationship with the School of Optometry and Vision Science or UNSW Sydney.

This research has been reviewed and approved by The University of New South Wales Human Research Ethics Committee. If you have any complaints or concerns about the research study, please email humanethics@unsw.edu.au or phone +61 2 9385 6222 quoting the following number iRECS7617.

Yours sincerely,
Prof. Fiona Stapleton
