# Supplementary material for: The effect of 0.1% topical ciclosporin a for 12-weeks on the ocular surface innate and adaptive immune response in dry eye disease: protocol for a randomized controlled clinical trial
Source: Ann Med. 2026 Sep 19;58(1):2729947. doi: 10.1080/07853890.2026.2729947 (PMC13625527; doi:10.1080/07853890.2026.2729947)
Supplement: Supplementary file 2 [file IANN_A_2729947_SM8080.pdf]

School of Optometry and Vision Science	UNSW SYDNEY

PARTICIPANT INFORMATION STATEMENT AND CONSENT FORM

The effect of 0.1% topical ciclosporin A for 12-weeks on immune cells at the surface of the eye in dry eyes

Scientia Professor Fiona Stapleton

1. **What is the research study about?**

You are invited to take part in this clinical trial research study. The study aims to investigate and compare the effect of 0.1% ciclosporin A (1mg/ml) eye drops with an ocular lubricant eye drop - mineral oils + glycerin for 12-weeks on the immune cells at the surface of the eye in dry eyes. We are conducting this clinical trial, to understand if this medication, ciclosporin A, can reduce immune cells on the eye surface better than using regular lubricating eye drops. Both ciclosporin and the lubricating eye drop are already approved for treating dry eyes and is listed in the Therapeutic Goods Administration (TGA) in Australia.

The researchers examining your eye and analysing your test results will not know which treatment you're receiving until after the study is complete. You will know which drops you are using, but the researchers analysing the results will not. This helps prevent bias in our measurements and analysis.

You will be randomly assigned (like flipping a coin) to one of two treatment groups:

Group A: You will receive: Ikervis (Australian Register of Therapeutic Goods ID 319502, containing ciclosporin A 0.1%) to use once daily at night, AND Cationorm (Australian Register of Therapeutic Goods ID 320453, a lubricating eye drop containing mineral oils and glycerin) four times each day.

Group B: You will receive: Cationorm (containing mineral oils and glycerin, a lubricating eye drop) four times each day.

This Participant Information Sheet/Consent Form (PISCF) informs you about the research study. It explains the tests and research involved so that you can decide if you would you like to participate in the research. Please read this information carefully. Ask questions about anything that you don’t understand or want to know more about.

2. Who is conducting this research?

The study is being carried out by the following researchers:

Role	Name	Organisation
**Chief Investigator**	Prof Fiona Stapleton	UNSW, School of Optometry and Vision Science
**Co-investigator**	Prof Isabelle Jalbert	UNSW, School of Optometry and Vision Science
**Co-investigator**	Prof Laura Downie	School of Optometry and Vision Science, University of Melbourne
**Co-investigator**	Prof Holly Chinnery	School of Optometry and Vision Science, University of Melbourne
**Student Investigator**	Mr. Soumen Sadhu	UNSW, School of Optometry and Vision Science
**Student Investigator**	Mr. Ali Alghamdi	UNSW, School of Optometry and Vision Science
**Student Investigator**	Mr. Farshid Karimi	UNSW, School of Optometry and Vision Science
**Co-investigator**	Dr Mengliang Wu	School of Optometry and Vision Science, University of Melbourne
**Co-investigator**	Ms Anne Lee	School of Optometry and Vision Science, University of Melbourne
**Co-investigator**	Ms Senuri Karunaratne	School of Optometry and Vision Science, University of Melbourne
**Research Funder:**	None	

3. Inclusion/Exclusion Criteria

Before you decide to participate in this research study, we need to ensure that it is okay for you to take part.

The research study is looking to recruit people who meet the following criteria:

- You are above 18 years of age
- You have signs and symptoms of or have already been diagnosed with dry eye disease.

You are not eligible to participate in the study if you fall under any of the following.

- You are currently using or have used any anti-inflammatory eye drops in the past 6 months.
- You are on oral ciclosporin or tacrolimus.
- You have known allergy to the study medication or any of its ingredients.

- You have an active eye disease inside the eye.
- You are a regular contact lens user or have used contact lenses in the last 4 weeks.
- You have an active eye infection or critical illness
- You have dry eyes due to Steven-Johnson syndrome, cicatricial conjunctival disease.
- You have other eye conditions or on medications for glaucoma.
- You have a history of eye surgery in the past 6 months.
- You are pregnant or breastfeeding.

4. Do I have to take part in this research study?

Participation in this research study is voluntary. If you do not want to take part, you do not have to. If you decide to take part and later change your mind, you are free to withdraw from the study at any stage.

If you decide you want to take part in the research study, you will be asked to:

- Read the information carefully (ask questions if necessary).
- Sign and return the consent form if you decide to participate in the study.
- Take a copy of this form with you to keep.

5. What does participation in this research require, and are there any risks involved?

If you agree to participate in this clinical trial, you will be asked to complete the following research procedures:

Eligibility screening: Your eligibility to participate in the study will be determined by taking a complete history, filling a questionnaire, and running some basic dry eye tests. The investigator will ask questions about your history of dry eye symptoms by telephone or in person. Your eligibility will be confirmed once you visit our clinic for the basic dry eye tests with your consent. The investigator will notify you if you are eligible or ineligible to participate in this study.

After determining the eligibility and signing the consent form, you will start a **washout period for 2-weeks,** where you will be given unpreserved lubricating eye drops to use in each eye up to four times daily. You will be asked to stop using any previous topical and oral medications

for dry eye. After the 2-weeks washout period, you will return to our clinic. You will be randomized (like flipping a coin) into one of the following groups

Group A – unpreserved Ciclosporin A 0.1% -Ikervis (containing ciclosporin A 0.1%) once daily at night-time + unpreserved Cationorm four times daily in both eyes for 12-weeks.

Group B – unpreserved Cationorm (containing mineral oils and glycerin) four times daily in both eyes for 12-weeks.

Ikervis is routinely prescribed in combination with unpreserved lubricating eye drops, such as Cationorm. The combination of these two medications in group A is a standard practice in eye care. There are no known or reported risks associated with the combination.

You will be given unpreserved unit dose ampules and bottles of eye drops involved in the study. During the treatment phase you will be asked not to use any other eye drops or oral medications for dry eye treatment. Both the eye drops are already approved for treating dry eyes and are listed in the Therapeutic Goods Administration (TGA) in Australia. The medications used in the study are commonly prescribed medications in Australia for the management of dry eyes, and we believe that you will have therapeutic benefit i.e., improvement of your dry eyes after using the medications in both groups.

The researchers examining your eye and analysing your test results will not know which treatment you're receiving until after the study is complete. You will know which drops you are using, but the researchers analysing the results will not. This helps prevent bias in our measurements and analysis.

After starting the therapy, you will visit our clinic for 2 follow-ups – at 4-weeks, and 12-weeks. The total duration for each visit will be around 1.5 hours.

You will undergo the following dry eye tests, and the discomforts and harms related to the study procedures are detailed below.

1**) Questionnaires (2 minutes)**: You will be asked to complete questionnaires related to dry eye symptoms: The Ocular Surface Disease Index questionnaire (OSDI); and the numerical rating scale (NRS) for symptoms questionnaire.

2) Tear Osmolarity (2 minutes): A disposable chip will be used to collect 50 microliters of tears from your eye. There is no harm in this procedure.

3) Keratograph 5M (5 minutes): This is an advanced corneal topographer with a built-in real keratometer and a colour camera optimized for external imaging. Unique features include examining the meibomian glands, non-invasive tear film break-up time and the tear meniscus height measurement. There is no harm in this procedure.

4) LipiView (2 minutes): The Lipiview (Tear Science INC, and Johnson and Johnson) a non-contact ocular surface interferometer will be used to measure the tear film lipid layer thickness. There is no harm in this procedure.

5) Tear collection (5 min): A paper strip will be placed under the lower eyelid for 5 min to collect tears. There is no harm in this procedure, but you might feel a mild discomfort for about 5-10 minutes in the lower eyelid area, where the paper strip was placed.

6) General eye check-up (2 minutes): You will undergo vision testing, examination of the front part of the eye (slit -amp examination). While the bright light source may cause mild discomfort to you, the light intensity can be adjusted.

7) Ocular surface evaluation (3 minutes): A wetted fluorescein strip (harmless yellow dye) and a wetted lissamine green strip (harmless green dye) will be then mildly touched to the lower eye lid. You will be asked to blink continuously to enable the smooth spreading of the stain over the eye, allowing for the evaluation of any surface defects. These are safe, and allergic reactions to the dye are uncommon. on. They occur in 1 in 1000 people, are mild and will go away without any treatment.

8) *In-vivo* confocal microscopy (45 minutes): A confocal microscope will be used to visualise the ocular surface epithelial immune cells and the corneal sub-basal corneal nerves. A probe that emits harmless light will come in contact with the transparent front part of the eye for approximately 1-2 minutes, and microscopic images of the cornea will be captured. Images will be captured from the right eye only. To avoid any discomfort the transparent surface of the eye will be numbed using an eye drop (anaesthetic).

Multiple images of the eye will be taken over a period of 30-45 minutes with 4-5 minutes multiple breaks in between. These regular breaks will allow you to relax, change position, and prevent discomfort from prolonged scanning. The procedure may induce some temporary discomfort and slight stinging for 10-20 minutes, which goes away without any treatment.

Procedures	Screening visit	Baseline	Follow-up visit 1. 4-weeks	Follow-up visit 2. 12- weeks
OSDI questionnaire	✓	✓	✓	✓
Numerical Rating Scale	✓	✓	✓	✓
Tear Osmolarity		✓		✓
Keratograph 5M	✓	✓	✓	✓
Lipiview II	✓	✓	✓	✓
In-vivo confocal microscopy		✓	✓	✓
Ocular surface evaluation	✓	✓	✓	✓
Impression cytology		✓		✓
Tear collection for inflammatory markers		✓		✓
Tonometry		✓	✓	

9) Impression cytology (5 min): This is a painless procedure to collect the eye surface cells. A small 12 mm in diameter filter paper will be applied firmly on the upper and lower part of the eye surface (conjunctiva). This will be applied for 2-4 seconds and will be taken out. You may experience a slight temporary discomfort during the procedure. Any discomfort induced would be expected to cease within 20 minutes after the procedure is completed.

10) Tonometry (2 minutes): This instrument is used to measure the eye pressure. While the eye is numb (anaesthetized) with eye drops, a probe gently touches the transparent part of the eye (cornea) for 5 seconds and the eye pressure is documented. There is no harm in this procedure.

What are the potential risks? We do not expect the study procedures or the eyedrops used in the study to cause any significant harm to you. You can drive safety immediately after the study procedures are conducted. The most common side-effects of both AND Cationorm are temporary and mild to moderate

- **i)** Eye irritation
- **ii)** Burning sensation
- **iii)** Watering from the eyes
- **iv)** Eye pain on eyedrop instillation.
- **v)** A rare side effect is severe allergic reaction to the eye to any of the compounds in the medications. If this occurs, you will stop all the study medications and proper care will be provided by our team.

The frequency of these side-effects is typically higher with ciclosporin in group A which are expected to occur around 13-37% of participants, whereas 8-22% of participants in group B as per previous studies.

Further, if you experience any discomfort or any feelings of distress because of participation in this study, you can let the research team know, and they will provide you with assistance. The Red Eye Clinic within UNSW Optometry Clinic is available with therapeutically endorsed optometrists to provide acute eye care. The UNSW Health Service is available to provide

medical care if required. You are free to withdraw from the research at any time; your decision will not affect your relationship with UNSW and the research team members. In an event of any study-related injury, you are covered by the UNSW clinical trial insurance and will receive treatment free of charge and will be eligible for further compensation. You will be monitored closely during the research period by the senior researchers and the research team. All discomforts and any harms will be documented and assessed. A study monitor will check periodically to ensure the study is conducted as per regulatory requirements.

**Name**	The Red Eye Clinic – UNSW Optometry Clinic
**Telephone**	+61 2 9385 6859
**Email**	optomclinic@unsw.edu.au

6. **What are the possible benefits of taking part?**

   The medications used in this study are approved in Australia to treat dry eye. You will be provided with all the study medications during the entire period of the study free of charge. You will receive free of cost study related care for your dry eyes, like cost-free study visits and dry eye tests.

   The study is important, as it will provide valuable information to us about the effect of these medicines tested in the study have on the eye immunity and that will improve dry eye therapy for patients like you. This will help us to develop new strategies and more informed dry eye therapies in the near future. **We also offer free parking for you in the university parking station.**

7. **Study Reimbursement**

   The name of all participants who agree to provide their contact details will enter a lucky prize draw for participating in the study. If you consent to provide your contact details, you will enter into a prize draw for participating in the study. **One $250 gift voucher will be awarded.** The prize draw will take place in July 2026, after study recruitment is completed. The winner will be notified via email using the contact details provided in the form.

8. What will happen to information about me?

By signing the consent form, you consent to the research team collecting and using information about you for the research study. The research team will store the data collected from you for this research project for a minimum of 15 years after the publication of the research results. The information about you will be stored in a re-identifiable format where any identifiers such as your name, address, date of birth will be replaced with a unique code.

You will be asked to provide your consent for the research team the share or use the information collected from you in future research that will be an extension of, or closely related to, the original project; or is in the same general area of research. You can decide not to let us use your data for future research by not checking the optional boxes on the consent form. Your information will only be shared in a format that will not identify you. Any data included in the reports or publications or presented at scientific meetings will be provided in the form of group responses and/or anonymously. Your personal and health information (either identifiable or potentially identifiable) will not be disclosed to any external parties without your consent unless required by law.

Information collected from you in an electronic format stored on a UNSW password protected OneDrive only accessible to the approved research investigators and backed up regularly onto the UNSW network server. Information collected from you using paper-based measures will be stored in the following locked filing cabinet at the School of Optometry and Vision Science and only the approved research investigators will have access to this information.

The information you provide is personal information for the purposes of the Privacy and Personal Information Protection Act 1998 (NSW). You have the right of access to personal information held about you by the University, the right to request correction and amendment of it, and the right to make a complaint about a breach of the Information Protection Principles as contained in the PPIP Act. Further information on how the University protects personal information is available in the UNSW Privacy Management Plan.

9. How and when will I find out what the results of the research study are?

The research team intend to publish and/ report the results of the research. All Information will be published in a way that will not identify you. If you would like to receive a copy of the results you can let the research team know by inserting your email or mailing address in the consent form. We will only use these details to send you the results of the research.

10. What if I want to withdraw from the research study?

If you do consent to participate, you may withdraw at any time. You can do so by completing the 'Withdrawal of Consent Form' which is provided at the end of this document, or you can ring the research team and tell them you no longer want to participate. Your decision not to participate or to withdraw from the study will not affect your relationship with UNSW. If you decide to leave the research study, the researchers will not collect additional information from you. You can request that any identifiable information about you be withdrawn from the research project.

11. What if I have a complaint or any concerns about the research study and will I receive compensation if suffer any injuries or have complications?

If you suffer any injuries or complications as a result of this research project, you should contact the study team as soon as possible and you will be assisted with arranging appropriate medical treatment. If you are eligible for Medicare, you can receive any medical treatment required to treat the injury or complication, free of charge, as a public patient in any Australian public hospital.

Complaints Contact If you have a complaint regarding any aspect of the study or the way it is being conducted, please contact the UNSW Human Ethics Coordinator:

**Position**	UNSW Human Research Ethics Coordinator
**Telephone**	+ 61 2 9385 6222
**Email**	humanethics@unsw.edu.au

**HC Reference Number**	N/A

12. What should I do if I have further questions about my involvement in the research study?

The person you may need to contact will depend on the nature of your query. If you require further information regarding this study or if you have any problems/discomfort which may be related to your involvement in the study, you can contact any one of the following member/s of the research team:

Clinic address: School of Optometry and Vision Science, Rupert Myers Building (M15), Southern Dr, UNSW, Kensington, NSW 2052

**Name**	Soumen Sadhu
**Position**	Student-Investigator
**Telephone**	+614 21892 071
**Email**	s.sadhu@unsw.edu.au

Research Team Chief Investigators Contact Details

**Name**	Prof Fiona Stapleton
**Position**	Chief-Investigator
**Telephone**	+61 418868234
**Email**	f.stapleton@unsw.edu.au
**Name**	Prof Isabelle Jalbert
**Position**	Co-Investigator
**Telephone**	+61 2 9065 7692
**Email**	i.jalbert@unsw.edu.au

If at any stage during the study, you become distressed or require additional support from someone not involved in the research please call support services. You can also contact a member of the research team and they will provide you with assistance, or alternatively, you can contact one of the support services

**Name/Organisation**	UNSW Optometry Clinic
**Position**	N/A
**Telephone**	+61 2 9385 4624
**Email**	optometryclinic@unsw.edu.au

or alternatively, **you can contact one of the support services**

**Mental Health Line**	1800 011 511
**Lifeline**	13 11 14

Consent Form – Participant providing own consent.

Declaration by the participant

- ☐ I understand I am being asked to provide consent to participate in this research study.
- ☐ I have read the Participant Information Sheet, or someone has read it to me in a language that I understand.
- ☐ I understand the purposes, study tasks and risks of the research described in the study.
- ☐ I provide my consent for the information collected about me to be used for the purpose of this research study only.
- ☐ I have had an opportunity to ask questions, and I am satisfied with the answers I have received.
- ☐ I provide my consent for the information collected about me to be used for the purpose of future research studies.
- ☐ I have had an opportunity to ask questions and I am satisfied with the answers I have received.
- ☐ I freely agree to participate in this research study as described and understand that I am free to withdraw at any time during the study and withdrawal will not affect my relationship with any of the named organisations and/or research team members.
- ☐ I understand that I will be given a signed copy of this document to keep.
- ☐ I would like to receive a copy of the study results via email or post, I have provided my details below and ask that they be used for this purpose only.
- ☐ I agree to provide my contact details.

Name: ____________________________________

Address: __________________________________

Email Address: _____________________________

Participant Signature

Name of Participant	
Signature of Research Participant	
Date	

Declaration by Researcher*

☐ I have given a verbal explanation of the research study, its study activities and risks and I believe that the participant has understood that explanation.

Researcher Signature*

Name of Researcher (please print)	Soumen Sadhu
Signature of Researcher	
Date	

+An appropriately qualified member of the research team must provide the explanation of, and information concerning the research study. All parties signing the consent section must date their own signature.

Participant providing consent for data to use for future research by the study researchers.

Declaration by the participant

- ☐ I give my consent to use my data for secondary study or future research by the study researchers.

Name: ____________________________________

Address: __________________________________

Email Address: _____________________________

Participant Signature

Name of Participant (please print)	
Signature of Research Participant	
Date	

Form for Withdrawal of Participation

I wish to **WITHDRAW** my consent to participate in this research study described above and understand that such withdrawal **WILL NOT** affect my relationship with The University of New South Wales.

- ☐ I am withdrawing my consent, and I would like any identifiable information collected about me which I have provided for the purpose of this research study withdrawn.
- ☐ I am withdrawing my consent for the information collected about me to be used for the purpose of future research studies.
- ☐ I am withdrawing my consent to participate in further components of this research and provide my permission for the research team to retain and/or use information collected about me which I have provided for the purpose of this research.
- ☐ I am withdrawing my consent, and I understand that any information already published and/or not linked to my identity cannot be withdrawn from the research.

Participant Signature

Name of Participant	
Signature of Research Participant	
Date	

The section for Withdrawal of Participation should be forwarded to:

CI Name:	Prof Fiona Stapleton
Email:	Chief-Investigator
Phone:	+61 2 9385 4375
Postal Address:	Rupert Myers Building (North Wing), School of Optometry and Vision Science, The University of New South Wales, UNSW SYDNEY NSW 2052 AUSTRALIA
